# Threats posed by stockpiles of expired pharmaceuticals in low- and middle-income countries: a Ugandan perspective

**DOI:** 10.2471/BLT.16.186650

**Published:** 2017-05-26

**Authors:** Pakoyo Fadhiru Kamba, Munanura Edson Ireeta, Sulah Balikuna, Bruhan Kaggwa

**Affiliations:** a Department of Pharmacy, School of Health Sciences, Makerere University, PO Box 7072, Kampala, Uganda.

## Abstract

In some low- and middle-income countries, the national stores and public-sector health facilities contain large stocks of pharmaceuticals that are past their expiry dates. In low-income countries like Uganda, many such stockpiles are the result of donations. If not adequately monitored or regulated, expired pharmaceuticals may be repackaged and sold as counterfeits or be dumped without any thought of the potential environmental damage. The rates of pharmaceutical expiry in the supply chain need to be reduced and the disposal of expired pharmaceuticals needs to be made both timely and safe. Many low- and middle-income countries need to: (i) strengthen public systems for medicines’ management, to improve inventory control and the reliability of procurement forecasts; (ii) reduce stress on central medical stores, through liberalization and reimbursement schemes; (iii) strengthen the regulation of drug donations; (iv) explore the salvage of officially expired pharmaceuticals, through re-analysis and possible shelf-life extension; (v) strengthen the enforcement of regulations on safe drug disposal; (vi) invest in an infrastructure for such disposal, perhaps based on ultra-high-temperature incinerators; and (vii) include user accountability for expired pharmaceuticals within the routine accountability regimes followed by the public health sector.

## Résumé

Dans certains pays à revenu faible et intermédiaire, les pharmacies nationales et les centres publics de santé détiennent d'énormes stocks de produits pharmaceutiques périmés. Dans les pays à revenu faible comme l'Ouganda, nombre de ces stocks proviennent de dons. Or, s'ils ne sont pas correctement contrôlés et réglementés, les produits pharmaceutiques périmés peuvent être reconditionnés et revendus en tant que contrefaçons ou bien jetés sans considération du danger pour l'environnement. Dans la chaîne d'approvisionnement, le pourcentage des produits pharmaceutiques périmés doit diminuer, et les produits périmés doivent être éliminés au bon moment et de façon sûre. De nombreux pays à revenu faible et intermédiaire doivent: (i) renforcer les systèmes publics pour la gestion des médicaments, afin d'améliorer le contrôle des stocks et la fiabilité des prévisions d'approvisionnement; (ii) réduire la pression à laquelle les pharmacies centrales sont soumises, grâce à des programmes de libéralisation et de remboursement; (iii) renforcer la régulation des dons de médicaments; (iv) étudier les options envisageables pour réemployer les produits pharmaceutiques officiellement périmés mais dont la durée de conservation pourrait éventuellement être prolongée après la réalisation de nouveaux tests; (v) renforcer l'application des réglementations pour une élimination sans risques des médicaments; (vi) investir dans des infrastructures d'élimination sans risques des médicaments, éventuellement au moyen d'incinérateurs à ultra-haute température; et (vii) responsabiliser les utilisateurs, en intégrant dans les programmes d'encadrement du secteur de la santé publique une obligation de rendre compte pour les produits périmés.

## Resumen

En algunos países con ingresos bajos y medios, los almacenes nacionales y las instalaciones del sector sanitario público contienen grandes cantidades de existencias de medicamentos que han superado su fecha de caducidad. En países con ingresos bajos como Uganda, muchas de estas existencias son el resultado de donaciones. Si no se controlan o regulan adecuadamente, puede ser que los medicamentos caducados sean empaquetados de nuevo y vendidos como falsificaciones o que se depositen sin tener conciencia del potencial daño medioambiental. Deben reducirse las tasas de caducidad de los medicamentos en la cadena de suministro y los medicamentos caducados deben depositarse a tiempo y de forma segura. Muchos países con ingresos bajos y medios necesitan: (i) fortalecer los sistemas públicos para la gestión de medicamentos para mejorar el control del inventario y la fiabilidad de la adquisición de provisiones; (ii) reducir el estrés en los almacenes médicos centrales a través de sistemas de reembolso y liberación; (iii) aumentar la regulación de las donaciones de medicamentos; (iv) investigar la recuperación de medicamentos oficialmente caducados a través de análisis y una posible extensión de la vida útil; (v) fortalecer la aplicación de regulaciones sobre la eliminación segura de medicamentos; (vi) invertir en una infraestructura para dicha eliminación, tal vez basada en incineradores de alta temperatura; e (vii) incluir la responsabilidad por parte de los usuarios en relación con los medicamentos caducados en los regímenes de responsabilidad rutinaria seguidos por el sector sanitario público.

## ملخص

في بعض البلدان منخفضة ومتوسطة الدخل، تحتوي المخازن الوطنية والمرافق الصحية التابعة للقطاع العام على مخزونات ضخمة من المنتجات الدوائية منتهية الصلاحية. أما في البلدان المنخفضة الدخل مثل أوغندا، فإن العديد من هذه المخزونات تكون ناتجة عن التبرعات. إذا لم تتم مراقبة المنتجات الدوائية منتهية الصلاحية أو تنظيمها بشكل كاف، يمكن إعادة تعبئتها وبيعها كأدوية مزيفة أو التخلص منها دون أي تفكير في الضرر البيئي المحتمل. ويلزم خفض معدلات انتهاء صلاحية الأدوية في سلسلة الإمداد والتوزيع، كما ينبغي التخلص من المنتجات الدوائية منتهية الصلاحية في الوقت المناسب وبطريقة آمنة على حد سواء. وتحتاج العديد من البلدان المنخفضة والمتوسطة الدخل إلى: (1) تعزيز النظم العامة لإدارة الأدوية لتحسين مراقبة المخزون وموثوقية توقعات المشتريات، و(2) تقليل الضغط على المخازن الطبية المركزية من خلال برامج التحرير والسداد، و(3) تعزيز تنظيم التبرعات الدوائية، و(4) التحقق من التخلص من المنتجات الدوائية منتهية الصلاحية رسميًا من خلال إعادة التحليل وإمكانية تمديد فترة الصلاحية، و(5) تعزيز إنفاذ اللوائح المتعلقة بالتخلص الآمن من الأدوية، و(6) الاستثمار في البنية التحتية للتخلص من الأدوية من خلال نظام قد يعتمد على أساس حرق المخلفات بالحرارة العالية، و(7) تضمين مساءلة المستخدمين عن المنتجات الدوائية منتهية الصلاحية في إطار نظم المساءلة الروتينية التي يتبعها قطاع الصحة العامة.

## 摘要

在一些中低收入国家，全国的商店及公共部门卫生机构拥有大量已超过其有效期的药品。 在乌干达等低收入国家，许多此类库存均来源于捐赠。 如果未给予充分监测或监管，过期药品则有可能进行重新包装并作为假冒产品进行销售或在不考虑任何潜在环境危害的情况下进行倾倒。 需减少供应链中药品的过期率并及时且安全地处理这些过期药品。 许多中低收入国家需： (i) 加强公共药物管理系统，以改善库存控制并提高采购预测的可靠性；(ii) 通过贸易自由化与补偿计划减少中央药店的压力；(iii) 加强药物捐赠监管；(iv) 通过再分析及合理延长有效期，探索已正式过期药品的挽救方法；(v) 加强安全药物处理条例的实施；(vi) 投资用于此类处理的基础设施，也许基于超高温焚烧装置；此外，(vii) 在公共卫生部门的日常问责制度范围内，纳入用户对过期药品的问责。

## Резюме

В некоторых странах с низким и средним уровнем дохода национальные склады и медицинские учреждения государственного сектора содержат огромные запасы фармацевтических препаратов, срок действия которых истек. В странах с низким уровнем дохода, таких как Уганда, многие такие запасы являются результатом пожертвований. При отсутствии надлежащего контроля или регулирования лекарственные средства с истекшим сроком годности могут быть переупакованы и проданы в качестве фальсификата или выброшены без какого-либо представления о потенциальном ущербе для окружающей среды. Частота случаев истечения срока годности лекарственных средств в цепи поставок должна быть снижена, а утилизация истекших фармацевтических препаратов должна проводиться как своевременным, так и безопасным способом. Многим странам с низким и средним уровнем дохода необходимо: (1) усовершенствовать государственные системы управления оборотом лекарственных средств, улучшить инвентарный контроль и надежность прогнозирования закупок; (2) уменьшить нагрузку на центральные медицинские склады за счет схем либерализации и возмещения расходов; (3) усилить регулирование пожертвований лекарственных средств; (4) изучить возможность сбора и дальнейшего использования фармацевтических препаратов с официально истекшим сроком годности путем повторного анализа и продления срока годности; (5) усилить контроль за соблюдением правил безопасной утилизации лекарственных средств; (6) инвестировать в инфраструктуру для такой утилизации на основе использования ультравысокотемпературных установок для сжигания отходов; (7) ввести ответственность пользователей за утилизацию фармацевтических препаратов с истекшим сроком годности в рамках установленных режимов подотчетности, которым следует сектор общественного здравоохранения.

## Introduction

In many low-income countries, and some middle-income countries, the government’s budget for the health sector is too small to finance the national health system adequately. In Uganda, for example, the expenditure on health in 2014 was only 12 United States dollars (US$) per capita, i.e. about 35% of the value recommended by the World Health Organization (WHO), and the expenditure on pharmaceuticals was just US$ 2.40 per capita.^1^ Such poor financing means that access to pharmaceuticals, like many other health sector priorities, has to be compromised. Many health systems have no choice but to rely, at least in part, on drug donations from high-income countries and vertical supplies from development agencies.^2^^,^^3^ During civil emergencies and periods of severe political instability, health systems may have to rely almost entirely on drug donations.^2^

Unfortunately, donated pharmaceuticals often mismatch the pharmaceuticals that are needed. International guidelines require that drug donations are responsive to the health needs of the recipient country and that the drugs involved have a shelf-life of at least one year on arrival.^4^ However, drugs that are already past their expiry dates have often been dumped in low- or middle-income countries^5^^–^^9^ and many past donations have been so large or so unwanted that they could not be used entirely before their expiry dates (Table 1).

**Table 1:** Examples of drug donations to low- and middle-income countries that did not appear useful, 1992–1999

Country	Problematic drug donation	Reference
Albania	Only 20% of donated drugs in 1999 were found useful	Bonn^5^
Bosnia and Herzegovina	Between 1992 and 1996, up to 60% of the 27 800–34 800 tonnes of medical supplies donated to what is now Bosnia and Herzegovina were not needed, resulting in 17 000 tonnes of pharmaceutical waste	McGregor^6^ and WHO et al.^9^
Djibouti	In 1994, only 12 co-trimoxazole tablets were found useful out of a large consignment of medicines donated to Médecins Sans Frontières, by a French nongovernmental organization	van der Heide and Schouten^8^
Georgia	In 1994, 20 tonnes of silver sulfadiazine ointment that was one year past its expiry date were given to an aid organization without notice and a large consignment of donated short-acting insulin arrived just 3 days before its expiry and, in 1995, there were 12 tonnes of unneeded drug donations – including 9 tonnes of expired drugs	Schouten^7^
Honduras	In 1998, many of the drug donations received were expired or close to expiry	Bonn^5^
Sudan	In 1990, large amounts of inappropriate drugs were received as donations	Bonn^5^
The former Yugoslav Republic of Macedonia	In 1999, more than 40% of drug donations were unneeded and about 30% arrived when expired or about to expire	Bonn^5^

WHO: World Health Organization.

Stockpiles of expired pharmaceuticals may also build up as a result of poor forecasts of future demand. Efficient stocking may be made difficult by deficiencies in the management of a supply chain or by poor coordination between a national supply system and the development partners or special programmes offering to supply pharmaceticals.^10^ In most low- and middle-income countries, the supply of pharmaceuticals is centralized and one state agency is entrusted with the procurement, storage and distribution of pharmaceuticals to all public health facilities.^3^^,^^11^ The network of public health facilities in any given country is often so expansive and complex that it is impossible for a single agency to respond effectively to the unique demands of each client. In Uganda in 2016, one central agency – the National Medical Store – was entrusted with supplying all pharmaceuticals to the country’s two national referral hospitals, 14 regional referral hospitals, 144 district hospitals, 197 county health centres, 1289 sub-country health centres and 2941 parish health centres.^12^ Unfortunately, few of these health facilities have staff members with the skills needed to manage pharmaceutical inventories or forecast future pharmaceutical needs effectively. In 2010, only 31 pharmacists were employed in Uganda’s public-sector health system.^11^^,^^12^ Structural and technical dysfunction in the management of a pharmaceutical supply chain can promote the accumulation of large quantities of expired pharmaceuticals in central stores and health facilities. In some low- and middle-income countries, including Uganda, civic observers and government oversight agencies have raised concern over the high incidence of expiry of stocked pharmaceuticals in the public supply system.^13^^–^^19^ The disposal of expired pharmaceuticals may also be a very slow process. In Uganda’s national medical store and public-sector health facilities, for example, such pharmaceuticals were found to be held for a mean of six years.^18^

In the absence of their timely and safe disposal, expired pharmaceuticals may be simply dumped – with the risk of environmental pollution – or repackaged for the counterfeit market. Toxicity from environmental exposure to pharmaceuticals has been reported in fish^20^ and vultures.^21^ If antibiotics are dumped, exposure to subtherapeutic concentrations of the drugs may lead to the selection of drug-resistant soil bacteria, which may then infect humans^22^^,^^23^ and even pass on their resistance genes to bacteria that are human pathogens.^24^ Mechanisms to deter the entry of pharmaceuticals into the environment need to be strengthened.

Below, we review the various options available for mitigating the threats posed by expired pharmaceuticals to health systems and the environment in low- and middle-income countries.

### Pharmaceutical pollution

The control of pharmaceutical pollution of the environment in low- and middle-income countries needs to be based not only on the safe disposal of expired drugs but also on the optimization of pharmaceutical use before expiry. New pharmaceutical expiries might be curtailed by: (i) strengthening the management of pharmaceutical supply chains in the public sector; (ii) reducing the workload at central medical stores, through liberalization and reimbursement schemes; (iii) improving the regulation of drug donation; and (iv) investigating the salvage of drugs that are officially expired but still usefully active, through re-analysis and possible shelf-life extension. There should be better supervision of stockpiles of expired pharmaceuticals and the disposal of such stockpiles needs to be improved and regulated better. The enforcement of any existing national and international regulations on the safe disposal of pharmaceuticals – e.g. by incineration at so-called ultra-high temperatures – needs to be strengthened. Every country needs to have such regulations.

### Management systems

Robust management systems for the supply of pharmaceuticals, in which re-order quantities are informed by reliable consumption data and demand forecasts, are essential in minimizing the amounts of pharmaceuticals that remain unused when they reach their expiry dates. In many low-income countries, it may be wise to invest in both robust information systems for logistics management, to track consumption, and the development of skilled human resources capable of optimizing forecasts of future demand. The use of computerized inventory management can greatly enhance data retrieval and analysis.^25^ In Uganda^12^ and wherever else that the unregulated pushing of pharmaceuticals to peripheral health facilities is often the norm, there needs to be a change to a demand-responsive system of supply. Oversupply, e.g. as a consequence of parallel procurements by several vertical health programmes,^10^ should be avoided by synchronizing the supply of all health commodities to public health facilities. Lastly, as unusual volatility in demand can reduce the turnover of inventory, channels for the redistribution of excess inventory to other public or even private health facilities^26^ should be strengthened.

### Central stores

To enhance the performance of publicly funded pharmaceutical supply, many low- and middle-income countries need to transfer some of the duties of the often overworked and stressed staff at central pharmaceutical stores to the staff at community pharmacies. Such a transfer might be supported by price-regulated, state-run reimbursement schemes or health insurance, as found in most high-income countries.^27^ At peripheral health facilities, where the capacity to track consumption and forecast demand is often inadequate, the delegation of prescription services to community pharmacies – wherever available – could again help to reduce the amounts of pharmaceuticals that remain unused when they reach their expiry dates.

### Drug donations

To suppress the unregulated export – from high-income countries to lower-income countries – of pharmaceuticals coming towards the end of their shelf-lives and other nonconforming medications, countries need to strengthen the enforcement of national policies and WHO guidelines on drug donations. According to WHO, any low- or middle-income country considering the receipt of a proposed drug donation should ensure that: (i) only solicited donations are allowed entry; (ii) any donated drug is approved for use in the recipient country and congruent with the relevant national policies and regulations; (iii) donations are in accordance with a plan mutually agreed upon by both the recipient and the donor; (iv) the donation is on the essential medicines list of the recipient country; and (v) the donation meets the quality standards of the donor and the recipient country.^28^ Ideally, the presentation of any donated medicine should match that already used in the recipient country, the labelling should be in a language that is widely understood in the recipient country and any donations of recycled medicines should be denied entry.^28^ Many low- and middle-income countries have designed their own policies, on the receipt of drug donations, that conform with WHO guidelines. For example, Uganda developed a national policy on drug donations in 1997. This policy not only adopted WHO recommendations but also added that any labelling and prescribers’ information should be in English and that details of the distribution and use of any donated drug must be sent, by the facility in which the drug was used, to the national medicines regulatory agency.^29^ In countries where there are no existing relevant laws and regulations, the national governments need to legislate on drug donations.

### Pharmaceutical salvage

Manufacturers generally assign pharmaceuticals shelf-lives of one to five years.^30^ Some pharmaceuticals are held in reserve for use in an emergency situation, such as an outbreak of an infectious disease, and many of these expire before any relevant emergency occurs. This can result in large stockpiles of expired pharmaceuticals, inventory losses and financial losses associated with stock disposal and replacement.^31^

To minimize the burden posed by the disposal and replacement of expired pharmaceuticals, the United States of America’s Food and Drug Administration has for more than three decades employed periodic testing and shelf-life review of pharmaceuticals that have good stability profiles. As a result of this initiative, which is known as the shelf-life extension programme, the shelf-lives of at least 88% of the tested products have been increased by at least one year.^30^^–^^32^

In low- and middle-income countries, it should be possible to extend the useful lives of medications that pass tests for efficacy and safety – and help save both money and the environment – in a similar manner. However, such shelf-life extension or drug salvage is only feasible where there is sufficient capacity for pharmaceutical analysis. In most low-income countries, any consideration of this intervention will have to be accompanied by discussion of investments in analytical infrastructure.

### Disposal

If unsafe disposal and leaching of pharmaceuticals into soil and water bodies are to be avoided, many low- and middle-income countries will have to strengthen the enforcement of national policies and WHO guidelines on pharmaceutical disposal. Within the WHO guidelines, it is recommended that: (i) the user unit should obtain approval for drug disposal from the appropriate authority, such as the national medicines regulatory agency; (ii) personnel at the disposal site should wear protective gear; (iii) expired pharmaceuticals are sorted into their different categories to ensure the appropriate disposal method is used for each category; and (iv) appropriate security is ensured during the disposal of controlled pharmaceuticals.^9^ Many low- and middle-income countries have adapted the WHO guidelines to their own situations. In Uganda, for example, the national medicines regulatory agency is the approving authority for medicines disposal. This agency has prescribed the steps to be taken and acceptable methods for the safe disposal of expired pharmaceuticals.^33^ Where none currently exists, national regulations on safe drug disposal ought to be formulated.

Although ultra-high-temperature incineration may be the most effective technique for the safe disposal of unwanted pharmaceuticals,^9^ it is not a cheap option^9^ and even the United States has found it hard to implement.^34^^–^^38^ Despite the perpetual burden posed by huge stockpiles of expired pharmaceuticals in the country, Uganda has only recently built its second ultra-high-temperature incinerator approved by the national medicines regulatory agency (D Nahamya, National Drug Authority, personal communication, 2017). In 2016, the costs of using these incinerators were high: an hourly supervisory fee, charged by the national medicines regulatory agency, of about US$ 30 plus a service fee, charged by the service provider, of US$ 0.75 per kg of pharmaceutical waste.^16^ The shortage of suitable incinerators and the high cost of using those that do exist promote the accumulation of pharmaceutical stockpiles. Given the threat to the environment posed by the unsafe disposal of pharmaceuticals, many low- and middle-income countries need to prioritize investments in ultra-high-temperature incineration and/or evaluate the safety of using cheaper methods of drug disposal – e.g. engineered landfill or waste immobilization by encapsulation or inertization.^9^

### Accountability

A potentially effective but rarely mentioned tool to prevent the misuse and improper disposal of expired pharmaceuticals is the enforcement of routine accountability for pharmaceuticals. For optimal effectiveness, low- and middle-income countries should make user accountability for expired pharmaceuticals part of the routine accountability regimes for their health sectors.

## Conclusion

As expired pharmaceuticals pose threats to both health systems and environments, low- and middle-income countries need to suppress the accumulation of such pharmaceuticals and their slippage into the environment or counterfeit drug markets. Critically, such countries need to strengthen the management of their pharmaceutical supply chains and the associated accountability and regulatory mechanisms.
